# Draft Genome Sequence of a Novel *Thermofilum* sp. Strain from a New Zealand Hot Spring Enrichment Culture

**DOI:** 10.1128/genomeA.00005-18

**Published:** 2018-02-22

**Authors:** Anna-Louise Reysenbach, John A. Donaho, Todd M. Hinsch, John F. Kelley, Kathleen Kouba, Mircea Podar, Matthew B. Stott

**Affiliations:** a Department of Biology, Portland State University, Portland, Oregon, USA; b Oak Ridge National Labs, Oak Ridge, Tennessee, USA; c Extremophile Research Group, GNS Science, Wairakei, New Zealand; d School of Biological Sciences, University of Canterbury, Christchurch, New Zealand

## Abstract

A draft genome of a new *Thermofilum* sp. strain was obtained from an enrichment culture metagenome. Like its relatives, *Thermofilum* sp. strain NZ13 is adapted to organic-rich thermal environments and has to depend on other organisms and the environment for some key amino acids, purines, and cofactors.

## GENOME ANNOUNCEMENT

Members of the crenarchaeal family *Thermofilaceae* (1) are widespread in terrestrial and marine hot springs (2, 3), with only two valid described species, *Thermofilum pendens* from Iceland (2) and *T. uzonense* from Kamchatka, Russia (3). Both have sequenced genomes (3, 4). Additional genomes are available for *Thermofilum* spp. from Kamchatka, “*T. adornatus*” 1910b (5) and “*T. carboxyditrophus*” 1505 (GenBank assembly accession number GCA_000813245). With the exception of “*T. librum*” (6), all *Thermofilum* spp. require *Thermoproteales* cell extracts or spent culture broth for growth. The extreme reduction in biosynthetic pathways for purines, amino acids, and cofactors in all *Thermofilum* genomes analyzed thus far suggests that these organotrophic thermophiles rely on other organisms and the environment for these small molecules. Here, we report the nearly complete draft genome of a new *Thermofilum* sp. strain, NZ13, obtained from a hot spring at Hell’s Gate (Tikitere) (38°03′47″S, 176°21′39″E; pH 6.0 and 74°C) in New Zealand.

Enrichments were maintained stably for 4 days at 80°C in a modified DSMZ medium (number 88) containing yeast extract (0.5 g/liter), tryptone (0.5 g/liter), and a headspace of hydrogen (100% vol/vol). DNA was extracted from the enrichment using the Qiagen DNAeasy blood and tissue DNA kit. Nextera DNA libraries were sequenced using an Illumina MiSeq instrument. Adaptors and low-quality reads were removed using Trimmomatic (7) and assembled using IDBA-UD (8). Contigs of less than 1 kb were removed. The contigs were binned using MaxBin (9), resulting in the nearly complete genome of *Thermofilum* sp. strain NZ13. Three additional bins for a *Fervidicoccus* sp., a *Desulfurococcaceae* sp., and a *Pyrobaculum* sp. were also generated. Optimization of the *Thermofilum* sp. NZ13 assembly was achieved by mapping the reads back using Bowtie 2 (10) and SAMtools (11, 12), reassembled using IDBA-UD, and further validated with Emergent Self-Organizing Maps (ESOM) (13). The draft genome (33 contigs) is 1.88 Mb and has 56.7% G+C content, which is higher than that of some *Thermofilum* genomes but similar to that of *T. pendens*. The genome completeness is about 99.3%, with very low (0.7%) contamination (CheckM [14]). The genome was annotated using Prodigal (15) and the arCOG database (16, 17). A total of 1,930 coding sequences, 47 tRNAs, and 5 clustered regularly interspaced short palindromic repeat (CRISPR) arrays were identified. Based on a RAxML concatenated phylogenetic tree of 16 ribosomal proteins and a 16S rRNA gene comparison using EzBioCloud (18) (98.4% 16S rRNA gene similarity to *T. uzonense*), *Thermofilum* sp. NZ13 probably represents a new species in the genus *Thermofilum*.

Like its relatives, the *Thermofilum* sp. NZ13 genome encodes numerous peptidases, glucosidases, and an alpha amylase, suggesting that the organism can grow on a range of peptides, polysaccharides, and starches. Additionally, *Thermofilum* sp. NZ13 shows a large reduction in biosynthetic genes, indicating that, similar to *T. pendens* and *T. uzonense*, it likely relies on the environment for most amino acids, purines, and cofactors. The genome encodes a large number of ABC transporter genes, with a high proportion being membrane transport proteins. This draft genome from a *Thermofilum* sp. from New Zealand illustrates that globally, species in this genus share very similar ecological niches.

### Accession number(s).

The *Thermofilum* sp. strain NZ13 draft genome data have been deposited in NCBI GenBank under accession number NDWX00000000. The version described in this paper is the first version, NDWX01000000.
